# Dynamics of Ultrathin Vanadium Oxide Layers on Rh(111) and Rh(110) Surfaces During Catalytic Reactions

**DOI:** 10.3389/fchem.2020.00707

**Published:** 2020-08-21

**Authors:** Bernhard von Boehn, Ronald Imbihl

**Affiliations:** Institut für Physikalische Chemie und Elektrochemie, Leibniz Universität Hannover, Hanover, Germany

**Keywords:** vanadium oxide, chemical waves, methanol oxidation, heterogeneous catalysis, oxide redistribution

## Abstract

Over the past 35 years rate oscillations and chemical wave patterns have been extensively studied on metal surfaces, while little is known about the dynamics of catalytic oxide surfaces under reaction conditions. Here we report on the behavior of ultrathin V oxide layers epitaxially grown on Rh(111) and Rh(110) single crystal surfaces during catalytic methanol oxidation. We use photoemission electron microscopy and low-energy electron microscopy to study the surface dynamics in the 10^−6^ to 10^−2^ mbar range. On VO_*x*_/Rh(111) we find a ripening mechanism in which VO_*x*_ islands of macroscopic size move toward each other and coalesce under reaction conditions. A polymerization/depolymerization mechanism of VO_*x*_ that is sensitive to gradients in the oxygen coverage explains this behavior. The existence of a substructure in VO_*x*_ islands gives rise to an instability, in which a VO_*x*_ island shrinks and expands around a critical radius in an oscillatory manner. At 10^−2^ mbar the VO_*x*_ islands are no longer stable but they disintegrate, leading to turbulent redistribution dynamics of VO_*x*_. On the more open and thermodynamically less stable Rh(110) surface the behavior of VO_*x*_ is much more complex than on Rh(111), as V can also populate subsurface sites. At low V coverage, one finds traveling interface pulses in the bistable range. A state-dependent anisotropy of the surface is presumably responsible for intriguing chemical wave patterns: wave fragments traveling along certain crystallographic directions, and coexisting different front geometries in the range of dynamic bistability. Annealing to 1000 K causes the formation of macroscopic VO_*x*_ islands. Under more reducing conditions dendritic growth of a VO_*x*_ overlayer is observed.

## Introduction

Chemical waves and kinetic oscillations on catalytic surfaces have been studied starting from about 1980 with the vast majority focusing on reactions on metal surfaces (Ertl, 1991; Imbihl and Ertl, 1995; Imbihl, 2005). Low pressure (*p* < 10^−3^ mbar) single crystal studies of catalytic CO oxidation and catalytic NO reduction on Pt, Rh, Pd and Ir catalysts yielded a plethora of different phenomena and they led to experimentally verified mechanisms. Despite the fact that the majority of the catalysts in chemical industry is based on oxidic materials, up to 2010 hardly any study of non-linear phenomena on oxidic catalysts existed. The reasons why metal catalyst were preferred against oxidic catalysts area easy to understand. Oxidic surfaces are structurally more complex, they are more difficult to prepare and surface analytical techniques based on electrons or ions are often of limited value due to beam damaging and electrical charging effects.

A concept to avoid these difficulties is based on the use of model systems in which ultrathin oxide layers are deposited on a metallic support. The main model system chosen here are submonolayer coverages of vanadium oxide deposited on a Rh(111) single crystal surface. Vanadium oxide based catalyst are widely applied in industrial catalysis, e. g., in partial oxidation reactions of hydrocarbons, in the selective catalytic reduction (SCR) in environmental catalysis, and in the production of sulfuric acid (Bond and Tahir, 1991). Important for the high catalytic activity is the ability of V to easily switch its oxidation state between +2 and +5. In most industrial applications, vanadium oxide catalysts are used in form of ultrathin layers, or as isolated clusters supported on an oxidic material of negligible catalytic activity. Of key importance is therefore knowledge about the dispersion of VO_*x*_ on a support material and redistribution dynamics under reaction conditions, which change the catalyst dispersion.

Besides the connection to “real catalysis,” the choice of the model system of supported submonolayer oxide films offers several advantages: (i) VO_*x*_ on Rh(111) exhibits a large number of ordered overlayers which have been exceedingly well characterized by surface analytical methods (Schoiswohl et al., 2004a,b; Surnev et al., 2013); for most of these overlayers structure models are available. (ii) Due to the electrically conducting support, electron-based analytical techniques can be applied. (iii) The system it strictly two-dimensional. Since V-oxides in their catalytically most active form are present either as isolated clusters or at monolayer coverages on a support material, studies of model catalysts with ultrathin layers of V-oxide are relevant to “real catalysis.”

The first aim of these studies was to see whether the spatially uniform distribution of the VO_*x*_ catalyst present after preparation is lifted under the influence of a catalytic reaction. Mostly the partial oxidation of methanol was chosen as reaction system, but also the H_2_ + O_2_ and other simple oxidation reactions were investigated. In fact, macroscopic stripe patterns and patterns of circular islands of VO_*x*_ developed under reaction conditions. The circular VO_*x*_ islands that developed exhibited a rather remarkable behavior. Two close-by islands moved toward each other and finally coalesced under reaction conditions (Hesse et al., 2015). Effectively, this leads to larger islands, similar to Ostwald ripening.

In order to see whether this type of behavior can be generalized, different reactions were investigated with VO_*x*_ on Rh(111) and, moreover, the Rh(111) substrate was exchanged against Rh(110) (von Boehn and Imbihl, 2018). The growth of VO_*x*_ on the more open and thermodynamically less stable surface Rh(110) surface is much more complex than on Rh(111) (von Boehn et al., 2017). In addition, this system had hardly been explored, so that no structure models were available. On the other hand, intriguing chemical wave patterns have been found in catalytic methanol oxidation on VO_*x*_/Rh(110), a behavior distinctly different from VO_*x*_/Rh(111) where only VO_*x*_ redistribution but no chemical wave patterns have been found. The results with both substrates, Rh(111) and Rh(110), are reviewed in this report.

Model catalyst studies and “real catalysis” cannot be connected without addressing the so-called “pressure and materials gap” problem in heterogeneous catalysis (Schlögl, 2015). The main strategy to close the pressure gap is the use of *in situ* or operando techniques, because only then one obtains the structure/composition of the active catalyst that is required for establishing a structure-function relationship. The low-energy electron microscope (LEEM) that found extensive use in this study is a typical UHV instrument designed to be operated close to 10^−10^ mbar. In an effort to bridge at least a large part of the pressure gap, this LEEM instrument was modified, so that it could be operated up to 10^−1^ mbar (Franz et al., 2019). Applied to catalytic methanol oxidation on Rh(111)/VO_*x*_, this modified instrument revealed that at 10^−2^ mbar the VO_*x*_ islands are no longer stable but disintegrate, displaying turbulent behavior (von Boehn et al., 2020).

One aim of this review is to summarize the progress that has been achieved in the study of non-linear phenomena on catalytic oxide surfaces using the example of ultrathin supported V-oxide layers. A second goal is to demonstrate the significance of non-linear dynamics for catalytic research. Quite impressive wave patterns have been found in catalytic reactions on single crystalline metal surfaces, but asked about the significance for catalysis, many scientists would be very reluctant to concede any significance at all. Several examples presented in this review, like VO_*x*_ redistribution patterns connected to an activation of the catalyst or reaction-induced ripening of oxide islands, demonstrate very convincingly that a coherent understanding of the properties of a catalyst is not possible without taking dynamic phenomena into account.

## Reaction Dynamics on VO_*x*_/Rh(111)

### Choice of Model System and Methods of Investigation

**Figure 1:**
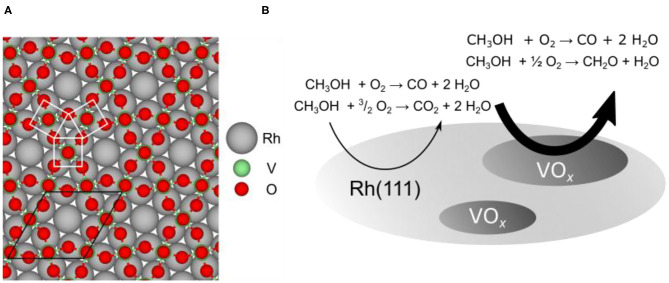
Structure and reactivity of VO_*x*_/Rh(111) model catalysts. **(A)** Structure model for the (√7 × √7)R19.1° vanadium oxide phase on Rh(111) (Schoiswohl et al., 2004b). The unit cell and a VO_5_ pyramid is indicated in the model. **(B)** Schematic representation of catalytic VO_*x*_ “microreactors” on Rh(111) during catalytic methanol oxidation. The main reactions on the bare Rh surface and vanadium oxide covered surface are indicated. Reprinted from von Boehn et al. (2020). Copyright (2020), with permission from Elsevier.

As a model system for studying the redistribution of VO_*x*_ catalysts during several catalytic reactions we utilize submonolayer VO_*x*_ films on Rh(111). The system VO_*x*_/Rh(111) has been extremely well characterized in the monolayer and submonolayer coverage range (Schoiswohl et al., 2004b, 2005, 2006). Numerous ordered overlayers exist and structure models are available for many of them. All these structures can be described as network structures following the same building principle: VO_*n*_ (*n* = 3–5) units are connected by sharing oxygen atoms (Schoiswohl et al., 2004b). An example of such a two-dimensional network structure, the so-called (√7 × √7)R19.1° structure, is shown in Figure 1A. The most abundant structural unit is a VO_5_ pyramid with a V atom being above the center of a square base formed by four O atoms (V atom hidden in Figure 1A by an O-atom on top of it).

This system exhibits several advantages: (i) the system is strictly two-dimensional; (ii) VO_*x*_ is highly mobile on the flat Rh(111) surface, (iii) submonolayer VO_*x*_ films on Rh(111) have a high catalytic activity in many oxidation reactions, and *iv)* charging problems are avoided thus allowing the use of electron-based surface analytical techniques. It should be added that the properties of ultrathin V-oxide layers can be markedly different from the properties of V-oxide bulk compounds (Surnev et al., 2013).

In industrial heterogeneous catalysis, typically metallic catalysts are dispersed on an oxidic support, e.g., SiO_2_, Al_2_O_3_, or TiO_2_. Here, oxides are deposited on a metallic support instead of a metal supported on an oxidic surface and, accordingly, the concept has been termed “inverse model catalyst” approach (Levin, 1987; Boffa et al., 1994; Surnev et al., 2013; Rodriguez et al., 2016). The concept of inverse model catalysts has several advantages. Charging problems with electron or ion based analysis techniques are avoided and the interface metal/oxide can be nicely studied. Moreover, the concept of inverse model catalysts has also been applied as a method to guide the development of novel catalysts (Zhang and Medlin, 2018).

In order to study the redistribution dynamics of VO_*x*_ spatially resolving techniques are required. In photoemission electron microscopy (PEEM) the sample surface is illuminated with UV light and the ejected photoelectrons are imaged (Rotermund, 1993). PEEM images primarily local work function variations with a lateral resolution of typically 0.1–1 μm. However, PEEM provides no or only indirect chemical and structural information. A technique that can provide the missing information is low-energy electron microscopy (LEEM) (Bauer, 2019) in its spectroscopic variant (SPELEEM) (Schmidt et al., 1998; Menteş et al., 2014). In LEEM, elastically backscattered, i.e., diffracted electrons in the energy range 0–200 eV are used to image the surface. Since the main contrast mechanism is diffraction contrast, LEEM is sensitive to structural changes of the surface, with the resolution being for most instruments around 10–15 nm, even though a resolution down to ~2 nm is possible (Wichtendahl et al., 1998). By putting in an aperture one can obtain diffraction images of an area as small as 1 μm diameter (μLEED). Similarly, at synchrotrons providing the X-ray photons, one can use the instrument to obtain X-ray photoelectron spectroscopy from a 1 μm spot (μXPS). One can thus characterize the local surface structure as well as the local chemical composition. Since this instrument combines the spatial resolution of a microscope with the ability to perform local spectroscopy, this technique has also been termed “spectromicroscopy.”

### Catalytic Activity and Dynamics of VO_*x*_/Rh(111)

In industrial partial oxidation of methanol, the “formox” process (Franz et al., 2010), formaldehyde is the desired product. In methanol oxidation over VO_*x*_/Rh(111) three catalytic reactions have to be considered, as schematically shown in Figure 1B. On the bare Rh(111) surface carbon dioxide, CO_2_, is formed as product of the total oxidation:

(1) $${\text{CH}}_{\text{3}}\text{OH }{\text{+3/2O}}_{\text{2 }}\to \;{\text{CO}}_{\text{2}}\text{ + }{\text{2H}}_{\text{2}}\text{O}.$$

Besides total oxidation, also carbon monoxide, CO, and formaldehyde, CH_2_O, are formed as products of the partial oxidation of methanol according to:

(2) $$\begin{array}{r} \text{C}{\text{H}}_{3}\text{OH }+{\text{O}}_{2\;}\to \text{CO }+\;2{\text{H}}_{2}\text{O}, \end{array}$$

(3) $$\begin{array}{r} \text{C}{\text{H}}_{3}\text{OH }+1/2{\text{O}}_{2\;}\to \text{ C}{\text{H}}_{2}\text{O }+\;{\text{H}}_{2}\text{O}. \end{array}$$

While CO is produced on both surfaces, bare Rh(111) and VO_*x*_ covered Rh(111), the formation of formaldehyde is restricted to the VO_*x*_ covered parts of the surface (Hesse et al., 2015).

The mechanism of methanol oxidation over oxide-supported V-oxide has been clarified in a number of quantum chemical studies (Döbler et al., 2005; Göbke et al., 2009; Kropp et al., 2014). More specifically, vanadyl groups (V=O) were shown to play an essential role in the reaction mechanism of formaldehyde formation from methanol over VO_*x*_ catalysts. Such vanadyl groups were found in some of the two-dimensional network structures of VO_*x*_ on Rh(111), for example in the (√7 × √7)R19.1° shown in Figure 1A (Schoiswohl et al., 2004b). In general, a mechanistic explanation of partial oxidation reactions over oxidic surfaces is often based on a two-step model originally proposed by Mars and van Krevelen (Frank et al., 2009; Carrero et al., 2014). First lattice oxygen from the oxidic catalyst is transferred to the reactant, which is oxidized. In a second step, the reduced oxide catalyst is reoxidized.

**Figure 2:**
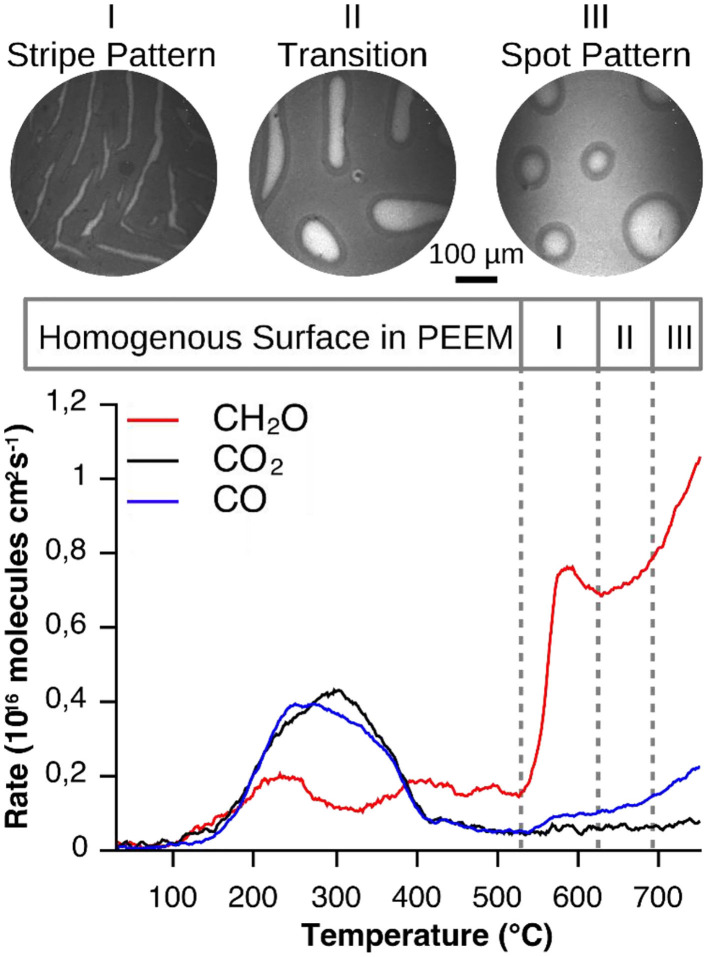
Vanadium oxide redistribution and reaction rates recorded during catalytic methanol oxidation over VO_*x*_/Rh(111). Experimental conditions: θ_V_ = 0.23 MLE, *p*(CH3OH) = 1 × 10^−4^ mbar, *p*(O_2_) = 1 × 10^−4^ mbar, 0.2°C/s. Reprinted figure with permission from Hesse et al. (2015). Copyright (2015) by the American Physical Society.

We investigate whether the initial homogeneous distribution of V-oxide is maintained during a catalytic reaction. The starting point of every VO_*x*_ redistribution experiment is a Rh(111) surface homogeneously, on a μm length scale, covered with a submonolayer quantity of VO_*x*_. It should be stressed that if the surface is homogeneous on a macroscopic scale (> 1 μm), on a microscopic scale it might be heterogeneous. Such a surface is exposed to a gas atmosphere consisting of methanol and oxygen in the 10^−4^ mbar range and heated to 1030 K (0.2–0.5 K/s), starting from 300 K. At around 770–820 K the initially homogeneous surface state is lifted and a regular stripe pattern develops, as shown in a series of PEEM images (stage I) in the top panel of Figure 2. In PEEM a low work function (WF) surface is imaged as bright area, high WF regions as dark area. Adsorbed oxygen, due to the high dipole moment of the adsorbate complex O-Rh, appears dark in PEEM, but so does V-oxide (if V is in a V^4+^ or V^5+^ state). Therefore, with PEEM alone, the interpretation of the images is not clear. Subsequent μXPS measurements (below) showed that the dark islands in Figure 2 represent V-oxide covered area, whereas the surrounding area is largely V-oxide free (Lovis et al., 2011; Hesse et al., 2015).

Remarkably, the condensation of the highly dispersed VO_*x*_ into a stripe pattern is accompanied by a drastic increase of the formaldehyde production (bottom panel of Figure 2). As the temperature is further increased the stripe pattern coarsens up to roughly 980 K (stage II), followed by a transformation into a spot pattern of circular, several tens to hundreds μm wide vanadium oxide islands (stage III).

**Figure 3:**
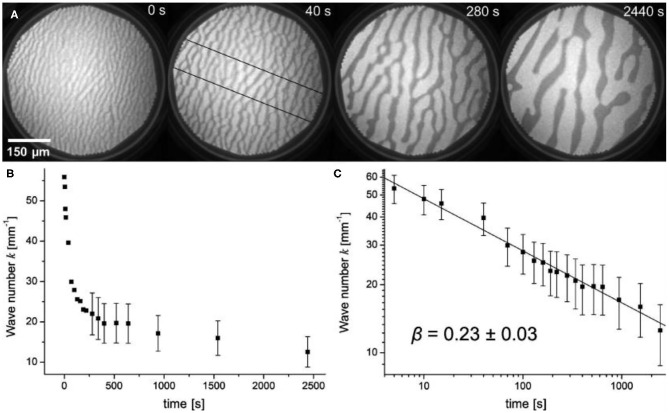
VO_*x*_ stripe pattern showing coarsening of a stripe pattern under constant reaction conditions in the O_2_ + H_2_ reaction on VO_*x*_/Rh(111). **(A)** Series of PEEM images showing the coarsening. **(B)** Plot of the wavenumber *k* as a function of time, determined along the cross sections indicated in the second image in a. **(C)** Double-logarithmic plot of *k* as a function of time. Experimental conditions: θ_V_ = 0.2 MLE, *p*(O_2_) = 1 × 10^−5^ mbar, *p*(H_2_) = 4 × 10^−6^ mbar. Reprinted with permission from Lovis and Imbihl (2011). Copyright (2011) American Chemical Society.

The formation of a VO_*x*_ stripe pattern followed by a transition into a spot pattern of VO_*x*_ islands is observed in a number of different chemical reactions on VO_*x*_/Rh(111), including the oxidation of H_2_, CO and ammonia (Lovis and Imbihl, 2011; von Boehn et al., 2016). Figure 3A shows the coarsening of a VO_*x*_ stripe pattern under constant reaction condition on VO_*x*_/Rh(111) during the H_2_ + O_2_ reaction at 773 K. The pattern is the result of V and O forming a dense VO_*x*_ coadsorbate phase. The surrounding bright surface is bare Rh(111) surface. The initially thin, dark stripes in the first PEEM image of Figure 3A increase their width, and after about 1000 s further progress is happening rather slowly. However, the coarsening still continues and we cannot deduce a value to which the curve asymptotically approaches.

This behavior is also reflected in a plot of the wavenumber *k* = 1/λ of the stripe pattern vs. time plot, as demonstrated in Figure 3B. The coarsening process obeys a power law *k* (*t*) ∞ *t*^−β^ with β = 0.23 ± 0.03, as shown in Figure 3C. For the dependence of the wavenumber *k* on the pressure, a power law *k*(*p*) ∞ *p*^α^ was experimentally determined with α = 0.33 ± 0.05. The wavenumber *k* increases with increasing pressure in the pressure range between 10^−6^ and 10^−4^ mbar.

For explaining pattern formation in this system, the concept of reactive phase separation seemed to be applicable. Attractive or repulsive interactions between adsorbed particles on a surface can result in the condensation into a dense phase. Thermodynamically, an island of infinite size should finally result but, coupled to a chemical reaction, a finite size is selected. On a potassium promoted Rh(110) surface reactive phase separation was observed during the H_2_ + O_2_ reaction (De Decker et al., 2004). Later on, reactive phase separation was also demonstrated on Pd and Au alloyed Rh(110) surfaces in the H_2_ + O_2_ reaction (Locatelli et al., 2005, 2006). The prerequisites for reactive phase separation to occur are not very restrictive, since only a sufficient mobility of the components and different chemical affinity between the reacting adsorbates are required. What does not fit into the concept of reactive phase separation in the present case is that the coarsening does not approach a finite value. A control experiment in which V-oxide on Rh(111) was annealed in O_2_ only, revealed that also in this case VO_*x*_ islands of macroscopic size are formed (von Boehn et al., 2016). The condensation of VO_*x*_ into stripes and circular islands under reaction conditions therefore appears to be driven by thermodynamics.

Under strongly reducing conditions, the reduction of VO_*x*_ can proceed until metallic V is formed, which forms surface and subsurface alloys with Rh(111) at sufficiently high temperature (1023 K) in vacuum (Píš et al., 2012). However, even low oxygen pressures in the 10^−7^-10^−8^ mbar range suffice to stabilize V as an oxide on the surface (Schoiswohl et al., 2005). Thus, under the conditions of catalytic reactions V is localized on the Rh(111) surface and no V/Rh surface or subsurface alloy is formed. This simplifies the analysis considerably.

### VO_*x*_ Islands Coalescence

#### Experimental Observations

**Figure 4:**
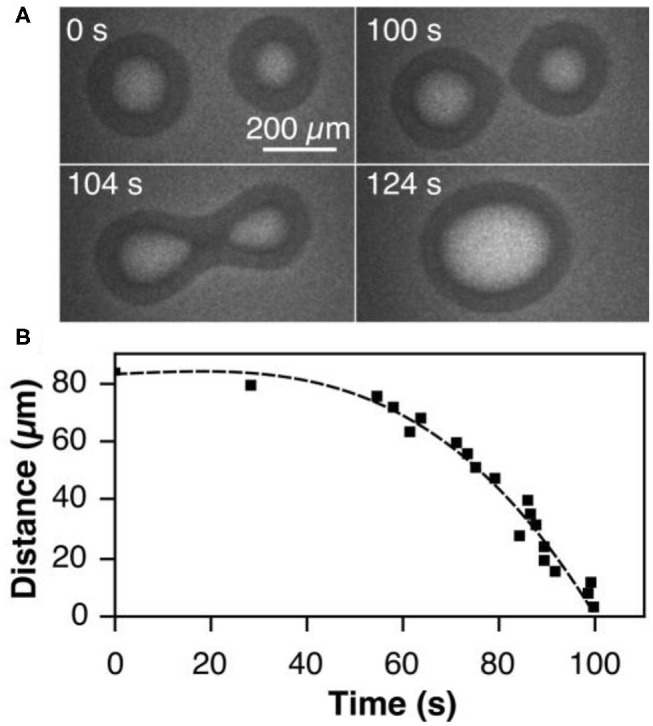
Coalescence of neighboring VO_*x*_ islands during catalytic methanol oxidation over VO_*x*_/Rh(111). **(A)** PEEM images showing different stages of the coalescence process. **(B)** Inter-island distance of the coalescence process shown in a as a function of time. Experimental conditions: θ_V_ = 0.23 MLE, *p*(CH3OH) = 1 × 10^−4^ mbar, *p*(O_2_) = 1 × 10^−4^ mbar, *T* = 750 °C. Reprinted figure with permission from Hesse et al. (2015). Copyright (2015) by the American Physical Society.

In the last stage of the self-organization process, stage III in Figure 2, circular tens to hundreds μm wide VO_*x*_ islands are distributed on the Rh(111) surface. Remarkably, if two V-oxide islands are within a critical distance of the order of 100 μm, they move toward each other and coalesce under reaction conditions. The series of PEEM images in Figure 4A shows the coalescence of two neighboring VO_*x*_ islands during catalytic methanol oxidation in the 10^−4^ mbar range. As the inter-island distance decreases, the movement of the two islands accelerates until they finally merge, as shown in Figure 4B. Interestingly, this coalescence proceeds exclusively under the conditions of an ongoing catalytic reaction. Stopping the supply of one of the two reactants, oxygen and methanol, instantly freezes the island movement.

One also notes that above a critical size the islands in Figure 4A exhibit a substructure consisting of an inner bright core, surrounded by an outer dark ring. This substructure is the result of V in the core being in a more reduced state than in the outer ring. This will be described below in more detail.

The island coalescence leads to an island ripening as in classical Ostwald ripening, where large islands grow at the expense of smaller ones. However, whereas Ostwald ripening occurs close to thermodynamic equilibrium, ripening via island coalescence takes place only under conditions far from equilibrium. The island coalescence mechanism is similar to Smoluchowski ripening, where island coarsening proceeds via collision of larger adatom aggregates, clusters or islands that diffuse randomly on the surface (Thiel et al., 2009). Smoluchowski ripening, however, does not require a chemical reaction, and the diffusing islands and clusters comprise only a small number of atoms. The unusual behavior seen here is the directed movement of large macroscopic islands that would normally require the existence of a macroscopic force.

#### The Polymerization/Depolymerisation Mechanism

For understanding the peculiar behavior of VO_*x*_ islands under reaction conditions, one has to take a closer look at the surface chemistry of V-oxides on Rh(111). Molecular oxygen has a low sticking coefficient on the VO_*x*_ covered Rh(111) surface, but it can readily adsorb on the metallic Rh(111) surface. As a consequence, O_ad_ is diffusively transported from the surrounding metallic Rh(111) surface toward the VO_*x*_ islands, where formaldehyde production almost exclusively takes place and where the oxygen is consumed. Due to the high catalytic activity of VO_*x*_, the macroscopic V-oxide islands can therefore be considered as “catalytic microreactors” which consume oxygen for the oxidation reaction, and thus act as sinks for adsorbed oxygen. The diffusive supply of O_ad_ to the VO_*x*_ islands generates gradients in the oxygen coverage around the V-oxide islands.

Observations with scanning tunneling microscopy on VO_*x*_ islands on Rh(111) demonstrated that at 380 K, isolated V_6_O_12_ clusters can detach from a large VO_*x*_ island (Schoiswohl et al., 2004a). If the cluster is part of a network structure in a VO_*x*_ island, additional oxygen is needed to detach the clusters, because in the network the individual units are connected by sharing corner oxygen atoms. If we take a V_2_O_3_ composition for the large VO_*x*_ islands, we can formulate a polymerization/depolymerisation (PD) or association/dissociation equilibrium:

(4) $$\begin{array}{r} {\left({\text{V}}_{2}{\text{O}}_{3}\right)}_{n}+3m{\text{O}}_{\text{ad}}\;⇌\;{\left({\text{V}}_{2}{\text{O}}_{3}\right)}_{n-3m}+m\left({\text{V}}_{6}{\text{O}}_{12}\right). \end{array}$$

**Figure 5:**
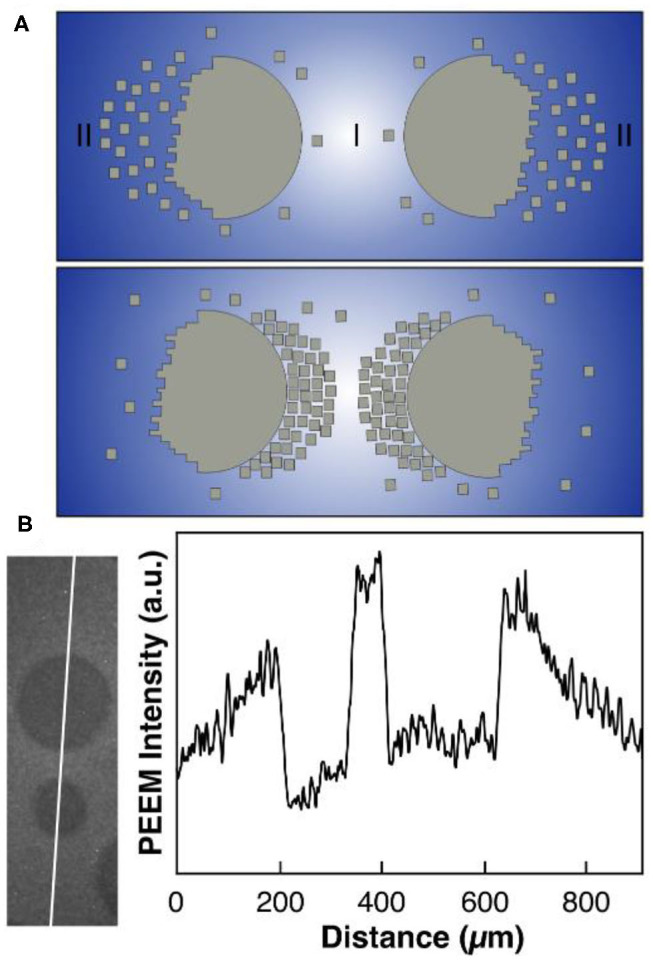
Polymerization/depolymerization mechanism explaining the coalescence of VO_*x*_ islands. **(A)** Scheme illustrating the movement of two neighboring VO_*x*_ islands via mobile V_*n*_O_*m*_ clusters (small squares) under reaction conditions. The intensity of the blue background represents the oxygen coverage. **(B)** Gradients in the oxygen coverage around VO_*x*_ islands extracted from PEEM. Experimental conditions: θ_V_ = 0.23 MLE, *p*(CH_3_OH) = 8 × 10^−5^ mbar, *p*(O_2_) = 1 × 10^−4^ mbar, *T* = 750 °C. Reprinted figure with permission from Hesse et al. (2015). Copyright (2015) by the American Physical Society.

This is a chemical equilibrium that is controlled by the oxygen coverage. A high oxygen coverage favors the dissociation of the large island into small clusters; conversely, a low oxygen coverage shifts the equilibrium toward aggregation of the clusters. The oxygen gradients developing under reaction conditions around VO_*x*_ islands and the PD equilibrium suffice to explain the island coalescence as schematically depicted in Figure 5. In region I in Figure 5A, the oxygen coverage is considerably lower than in region II, since two neighboring VO_*x*_ islands compete as sinks for adsorbed oxygen. According to the PD equilibrium of Equation (4), a high oxygen coverage in regions II favors depolymerization of the VO_*x*_ islands into small V_6_O_12_ clusters. Since the low oxygen coverage in region I shifts the equilibrium to the polymerization side, the small V_6_O_12_ clusters become attached again to the VO_*x*_ islands, but now on the opposite side from where they became detached. The net result is that the two neighboring islands move toward each other.

An essential requirement of this mechanism is that the V_6_O_12_ clusters and adsorbed oxygen exhibit a sufficiently high diffusivity. Both is the case as shown by experimental data (Barth, 2000; Mavrikakis et al., 2002; Schoiswohl et al., 2004a). Experiments in which VO_*x*_ islands pinned to defects “evaporated” demonstrated the existence of small mobile VO_*x*_ clusters, because desorption of VO_*x*_ into the gas phase can be excluded (Hesse et al., 2015). The diffusivity of atomic oxygen on Rh(111) fixes the length scale of the oxygen gradients, and this value of roughly 100 μm is equal the critical distance below which two neighboring islands start to feel the attraction of the other island. The existence of oxygen gradients around VO_*x*_ islands shows up in brightness variations in PEEM (Figure 5B) and, more directly, in concentration profiles recorded with μXPS (below). The mechanism sketched above explains why island movement only occurs under reaction conditions since, without reaction, the oxygen gradients immediately vanish. Clearly, no macroscopic force is needed to move the islands because the movement takes place via the transport of small V_*n*_O_*m*_ clusters.

#### Modeling VO_*x*_ Islands Coalescence

The island coalescence in the system CH_3_OH + O_2_/VO_*x*_/Rh(111) has been reproduced with a skeleton reaction-diffusion model (De Decker et al., 2019). This model considers a highly simplified reaction mechanism, but takes into account the PD mechanism including the adsorption of oxygen and the existence of energetic interactions between the adparticles. Strong lateral interactions between the VO_*x*_ polymers are evidenced by the nearly circular shape of the VO_*x*_ islands, which indicates a strong internal cohesion.

The small V_*n*_O_*m*_ clusters through which VO_*x*_ mass transport is mediated are represented as “monomers” M in the model, extended V-oxide areas are described as “polymer” P. The polymerization / depolymerization equilibrium in Equation (4) can then be written as:

(5) $$\text{P + M + }\left[*,{\text{O}}_{\text{ad}}\right]\;⇌\text{ 2P + }{\text{O}}_{\text{ad}},$$

with $\left[*,O_{ad}\right]$ representing an unoccupied adsorption site for *O*_*ad*_.

Adsorbed oxygen is formed by dissociative adsorption from the gas phase,

(6) $$\begin{array}{l} {\text{O}}_{\text{2}\left(\text{g}\right)}\text{+ 2 }\left[*,{\text{O}}_{\text{ad}}\right]\;⇌\text{ 2 }{\text{O}}_{\text{ad}},\\ \; \end{array}$$

and through the condensation process of monomers described in Equation (5). Oxygen is removed through desorption and through reaction with a molecule X (e.g., methanol) according to

(7) $${\text{O}}_{\text{ad}}\text{ + }{\text{X}}_{\text{ad}}\;\to \;{\text{XO}}_{\left(\text{g}\right)}\text{+ }\left[*,{\text{O}}_{\text{ad}}\right].$$

As outlined above, this reaction proceeds with a considerable higher rate on the vanadium oxide islands (catalytic “microreactors”) than on the bare metal surface. The different catalytic activity is taken into account via the rate constant of the catalytic reaction, which is a function of the local polymer coverage θ_P_.

The surface diffusion of atomic oxygen (*D*_O_), of the monomer M (*D*_M_), and of the polymer P (*D*_P_) are taken into account with *D*_O_ > *D*_M_ >> *D*_P_. Due to the existence of energetic interactions between adsorbed particles and due to site exclusion, diffusion is non-Fickian.

Numerical integration of the reaction-diffusion-equations shows that a region of bistability exists in which the homogeneous distribution of VO_*x*_ decays spontaneously into a P-rich and a P-poor phase in a quasi-spinodal decomposition. The domain size does not approach an asymptotic value in time but continuously increases as experimentally observed in the H_2_ + O_2_ reaction on VO_*x*_/Rh(111) (Figure 3).

**Figure 6:**
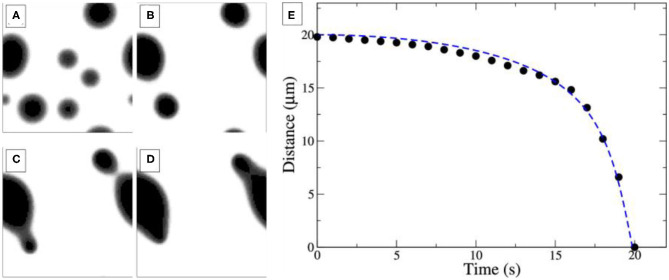
Simulations of coalescing VO_*x*_ islands on Rh(111). The system size is 50 × 50 μm^2^. **(A–D)** Four snapshots taken at *t* = 0 s **(A)**, 30 s **(B)**, 80 s **(C)**, and 130 s **(D)**, with *t* = 0s corresponding to the time when reaction conditions are turned on. **(E)** Inter-island distance. For details and simulation parameters please refer to the original publication. Adapted with permission from Makeev and Imbihl (2019). Copyright (2019) American Chemical Society.

As one increases both, the reaction rate and the O_2_ adsorption rate, which in experiments corresponds to elevated temperature and pressure, one observes the behavior depicted in Figure 6. Close-by islands move toward each other and coalesce. Directly after coalescence the islands are deformed, but the circular shape is restored within a short time. The distance between the two coalescing islands plotted vs. time in Figure 6E displays qualitatively the same behavior as the experimental dependence in Figure 4B. A close look at the concentration profiles corroborates the mechanistic interpretation given above. It is the sensitivity of the PD equilibrium to gradients in the oxygen coverage that leads to the transport of VO_*x*_ and causes the movement of the VO_*x*_ islands toward each other.

The reaction-diffusion equations allow even to derive an equation of motion for the islands using Newtonian mechanics. Numerical integration of this Newtonian law reproduces well the trajectories obtained from simulations of the full model, as demonstrated in Figure 6E (dashed blue line).

#### VO_*x*_ Island Substructure

**Figure 7:**
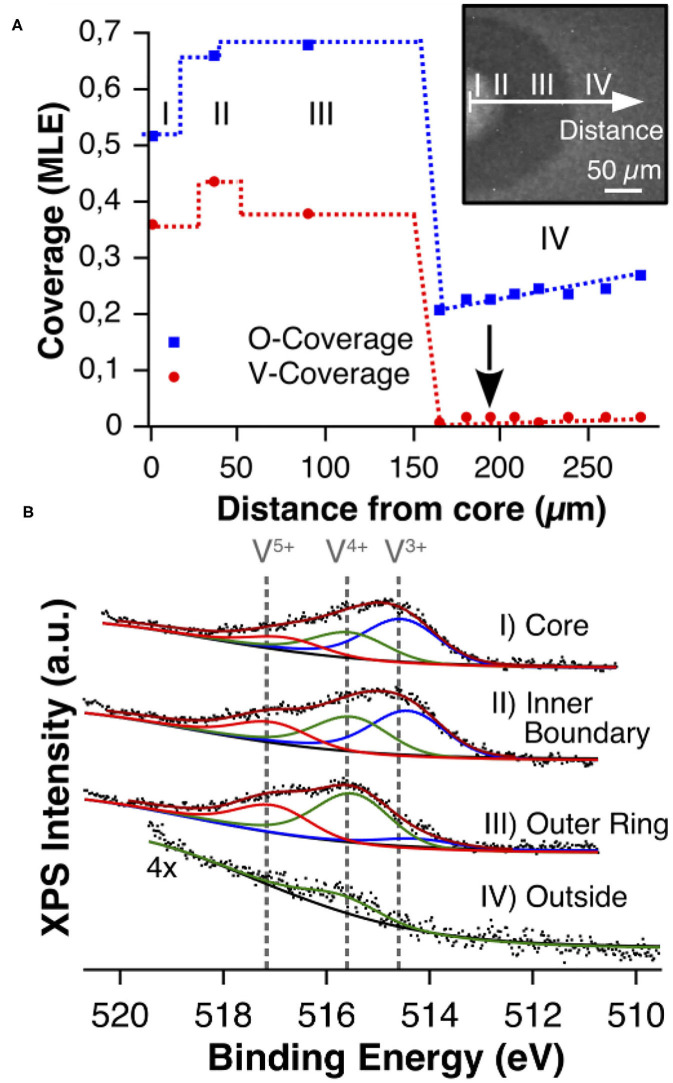
Chemical characterization of a VO_*x*_ island with XPS during catalytic methanol oxidation over VO_*x*_/Rh(111). **(A)** V and O coverage profile recorded under reaction conditions in the 10^−6^ mbar range. The position 0 μm refers to the island center. **(B)** V 2p_3/2_ core level spectra recorded with μXPS on various positions inside and outside (arrow) the VO_*x*_ island. Reprinted figure with permission from Hesse et al. (2015). Copyright (2015) by the American Physical Society.

The PEEM images in Figures 2, 4A show that the VO_*x*_ islands exhibit a substructure consisting of a bright core and a dark outer ring. The missing chemical information is provided by μXPS, allowing also for quantification of the surface coverages. After preparation of a VO_*x*_ island in the 10^−4^ mbar range, local XP spectra were taken under reaction conditions in the 10^−6^ mbar range across the island (Hesse et al., 2015). One thus obtains a cross section through the island displayed in Figure 7A. A shift of the V 2p core level toward higher binding energy (BE) in Figure 7B indicates that vanadium in the outer ring is in a more oxidized state than in the core region. A comparison of the V 2p BEs from the spectra with the BEs from V-oxide bulk compounds (neglecting possible effects from the monolayer thickness), shows that in the center of the island V^3+^/V^4+^ species prevail, whereas in the outer ring V is mainly in the oxidation state +4/+5. This finding agrees with the picture of VO_*x*_ islands as “microreactors,” because with oxygen being transported diffusively from the island boundary, naturally an oxygen concentration gradient will arise inside the island caused by oxygen consumption in the reaction. Above a critical island size, the supply of oxygen will not be high enough to maintain a high oxidation state of vanadium in the core region of the island, and a reduction through reaction with methanol will occur.

As expected from the picture of the “microreactor,” the oxygen coverage is high outside the VO_*x*_ islands and a gradient is clearly visible from a high oxygen coverage far away from the island toward a lower coverage at the boundary. Outside the VO_*x*_ islands, only a small amount of vanadium oxide is detected, representing the mobile V_*n*_O_*m*_ clusters through which the island coalescence proceeds.

Besides the island radius, the existence of a substructure also depends on the reaction conditions. A high amount of methanol favors the formation of a reduced core. One should add that VO_*x*_ islands can be prepared with a substructure involving a number of clearly distinguishable phases (von Boehn, 2020). The effect of the substructure on the dynamics of an island is demonstrated in the following.

### Oscillating VO_*x*_ Islands

**Figure 8:**
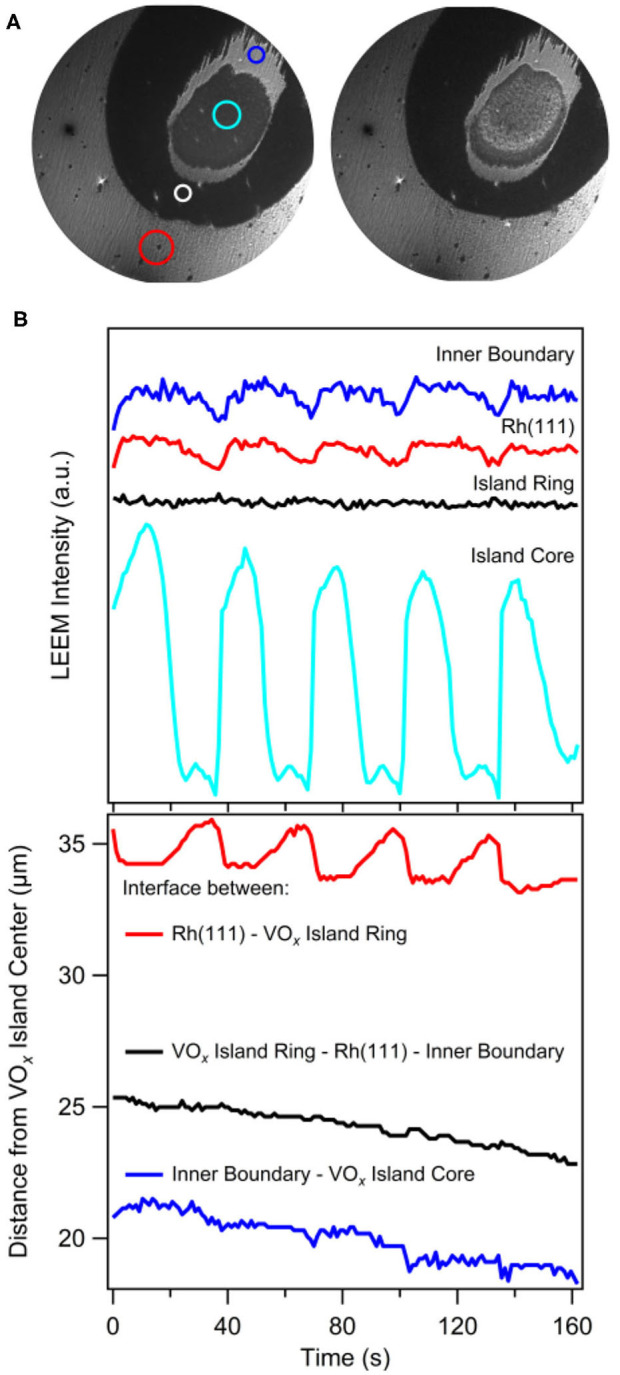
Oscillating VO_*x*_ islands during methanol oxidation on VO_*x*_/Rh(111). **(A)** Two LEEM images showing a circular VO_*x*_ island with the substructure consisting of a core and a ring, with the core being in its oxidized (left) and its reduced (right) state. **(B)** Local LEEM intensity (taken from areas indicated by colored circles in a) (top) and the position of the different interfaces (bottom) as a function of time. Experimental conditions: 2 eV electron energy, 1 × 10^−4^ mbar CH_3_OH and 1 × 10^−4^ mbar O_2_, *T* = 1030 K. Reprinted from von Boehn et al. (2020). Copyright (2020), with permission from Elsevier.

Two subsequent LEEM images showing two stages in the oscillatory behavior of a circular VO_*x*_ island during catalytic methanol oxidation are shown in Figure 8A. Reaction conditions are in the 10^−4^ mbar range. Different from PEEM, whose main contrast mechanism is based on local work function variations, LEEM is a structure sensitive microscopy technique, which additionally allows for laterally resolved LEED measurements. The brightness levels in LEEM are not related to the brightness levels in PEEM, since the contrast mechanisms are different. In the LEEM images one observes a core region, a dark outer ring and, in addition to the substructure visible in PEEM, a band separating core and outer ring.

Surprisingly, despite constant parameters, the islands exhibits an oscillatory cycle in which the island undergoes a periodic expansion and contraction, simultaneously with periodic brightness variations in the core of the island (von Boehn et al., 2020). The transformations from a bright core to a dark core and vice versa take place via reaction fronts nucleating at the boundary of the core. The periodic LEEM intensity changes of the different VO_*x*_ phases as well as the changes in the positions of the three interfaces are displayed in Figure 8B. Practically only the outer ring changes its size and the size oscillations display a strict phase relationship with the periodic intensity changes in the core that we can associate with a reduction and oxidation, respectively. A remarkable fact is that in this “breathing” of the island a considerable mass transport of VO_*x*_ has to occur to accomplish the variations in island size.

With *in situ* μLEED ordered VO_*x*_ overlayers could be identified in the breathing island: a (√7 × √7)R19.1° structure for the outer ring and a (√3 × √3)-Moiré structure for the core region (von Boehn et al., 2020). To understand the occurrence of different phases in the VO_*x*_ island is quite straightforward using the picture of the VO_*x*_ islands as catalytic micro-reactors. Since inside the oxide islands a gradient in the chemical potential of oxygen exists, a cross section through a VO_*x*_ island corresponds to a cross section through a phase diagram of VO_*x*_ on Rh(111), with the chemical potentials of oxygen and vanadium as intensive variables.

**Figure 9:**
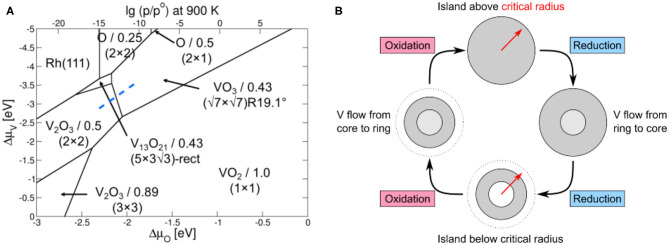
Mechanism explaining the VO_*x*_ island oscillations shown in Figure 8. **(A)** DFT phase diagram of the 2D VO_*x*_ phases on Rh(111). The VO_*x*_ structures and corresponding coverages are given. The transition between an oxidized phase and a reduced phase is indicated by the dashed blue line. Reprinted from von Boehn et al. (2020). Copyright (2020), with permission from Elsevier. **(B)** Sketch of a tentative mechanism explaining the periodic, “breathing-like” expansion and contraction of a vanadium oxide island. Reprinted with permission from von Boehn (2020).

Such a phase diagram has been calculated with density functional theory (DFT) for the system VO_*x*_/Rh(111) and the result is displayed in Figure 9A. The (√7 × √7)R19.1° structure of the highly oxidized ring is present, but the (√3 × √3)-Moiré structure of the core does not occur in the phase diagram. This (√3 × √3)-Moiré structure has not been reported in the literature and no structure model exists. Probably, this structure does not represent an equilibrium structure and therefore does not show up in a phase diagram of VO_*x*_/Rh(111). Since V in the reduced core is present as V^3+^, a (2 × 2) V_2_O_3_ structure with a V coverage of 0.5 MLE is predicted from the phase diagram for the reduced island core.

The phase diagram reveals that the reduced VO_*x*_ phases have a higher V density than oxidized VO_*x*_ phases, a relation that also became apparent from the experimentally determined V and O coverages for the different 2D-VO_*x*_ phases on Rh(111) (Schoiswohl et al., 2004b, 2005; von Boehn et al., 2020). For explaining the size oscillation, only one other relation is required. Since periodic oxidation/reduction fronts transform the core of the island, the island size has to be in the vicinity of the critical radius for existence of a reduced core. With these two ingredients, *i)* different V densities for reduced and oxidized VO_*x*_ phase, and *ii)* the island size being around the critical radius for the formation of a reduced core in VO_*x*_, we can set up a mechanism for the size oscillations. We assume that in the oxidation/reduction of the island core, we move along the blue dashed line in the PD of Figure 9A.

A Schematic Representation of the Proposed Mechanism is Shown in Figure 9B:

- Initially, the island is above the critical island radius, but the core is still in a completely oxidized state. Due to the long distance over which O_ad_ has to diffuse, not enough oxygen reaches the core, and a reduction of the core sets in. Since the reduced VO_*x*_ phase has a higher V density, extra V atoms are needed. They are taken from the outer ring and are transported from there to the island center.
- The transport of V atoms to the island core results in a shrinking of the oxidized ring, which eventually falls below the critical radius.
- Due to the shorter distance O atoms now have to cover, the O coverage in the island core increases, finally triggering the transformation of the core back into the oxidized state.
- Due to the lower V density in the oxidized state, extra V atoms are expelled from the core. They migrate back to the outer ring, which grows thus closing the oscillatory cycle.

The oscillatory change of the VO_*x*_ island size requires that a mass transport of vanadium is involved in addition to the transport of oxygen. According to the Onsager relations, a gradient in the chemical potential of oxygen is connected with a corresponding gradient in the chemical potential of vanadium (Mikhailov and Loskutov, 1991; Mikhailov, 1994). Clearly, a thermodynamic driving force for the transport of V therefore exists, but it is not clear in what form V is transported, since no experimental data exist for the diffusion inside VO_*x*_ islands. V mass transport within the island may occur via diffusion of isolated V atoms or via V-oxo complexes.

### Bridging the Pressure Gap

The existence of a pressure gap in heterogeneous catalysis means that the results obtained at low pressure cannot, in general, be extrapolated to reaction conditions at higher pressure. In order to bridge the pressure gap, a variety of *in situ* and operando techniques have been developed in order to characterize a catalyst as close as possible to realistic conditions (Topsøe, 2003; Dou et al., 2017). The LEEM is a typical ultrahigh-vacuum (UHV) instrument designed for operating at 10^−10^-10^−8^ mbar. The operational range of LEEM has recently been extended up to 0.1 mbar, a range also denoted as near-ambient-pressure (NAP) range (Franz et al., 2019). With NAP-LEEM, the redistribution dynamics of VO_*x*_ catalysts on Rh(111) could be followed up to the 10^−2^ mbar range (von Boehn et al., 2020).

**Figure 10:**
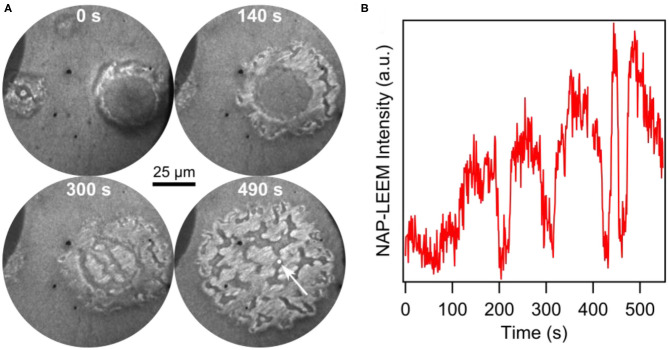
Transition of a VO_*x*_ island from a core-ring structure to turbulent dynamics during catalytic methanol oxidation over VO_*x*_/Rh(111) at 10^−2^ mbar. **(A)** Series of NAP-LEEM images (3.1 eV) recorded between 650 and 670 K. The time elapsed since the beginning of the island disintegration is indicated. **(B)** Local LEEM intensity as a function of time taken in the circular white area of 3 μm diameter (white arrow in the last image of **(A)**. Reprinted from von Boehn et al. (2020). Copyright (2020), with permission from Elsevier.

After deposition of 0.2 MLE V in vacuum, the pressure was raised into the 10^−2^ mbar range. The reaction dynamics of methanol oxidation over VO_*x*_/Rh(111) was followed with NAP-LEEM during heating up, as shown by the NAP-LEEM images in Figure 10A. Circular VO_*x*_ islands with the familiar core—ring structure form. As the temperature is raised beyond 650 K, this structure starts to disintegrate beginning at the outer ring. A slight island expansion is followed by the nucleation and growth of bright areas in the outer ring. Simultaneously, additional dark, channel-like structures develop. At slightly higher temperature, a bright phase nucleates also in the island core. After a few seconds the original core-shell structure of the VO_*x*_ island is gone and the area is covered by bright and dark phases that continuously change. The dark, channel-like areas meander on the surface, forming long extensions, which often break and reconnect with other parts of the dark phase. A time series of the local NAP-LEEM intensity displayed in Figure 10B demonstrates irregular behavior. The visual impression is that of a turbulent redistribution dynamics.

Due to the lack of surface analytical techniques that can operate *in situ* in the 10^−2^ mbar range, no further chemical or structural information is available. Obviously, a stable VO_*x*_ distribution on the support surface cannot be maintained in the 10^−2^ mbar range, as evidenced by the turbulent redistribution dynamics. At a pressure of 10^−2^ mbar, gradients in the oxygen coverage are presumably stronger than at low pressure, which might be a cause for the lack of stability in the island structure. Furthermore, in the NAP regime, the reaction conditions can no longer be considered as strictly isothermal, as it is the case under vacuum conditions up to roughly 10^−3^ mbar.

### Oxidation With NO

One of the first questions that were asked after catalytic methanol oxidation exhibited quite remarkable reaction dynamics on VO_*x*_/Rh(111), was how general the island coalescence mechanism is. Experiments in which methanol oxidation was replaced by the H_2_ + O_2_ reaction, the NH_3_ + O_2_ reaction, and catalytic CO oxidation on VO_*x*_/Rh(111) showed in fact qualitatively the same behavior as in catalytic methanol oxidation (von Boehn et al., 2016). However, all these reactions have in common that O_2_ is used as oxidizing agent. Replacing O_2_ by NO as oxygen source leads in fact to a quite different behavior (von Boehn et al., 2018a).

**Figure 11:**
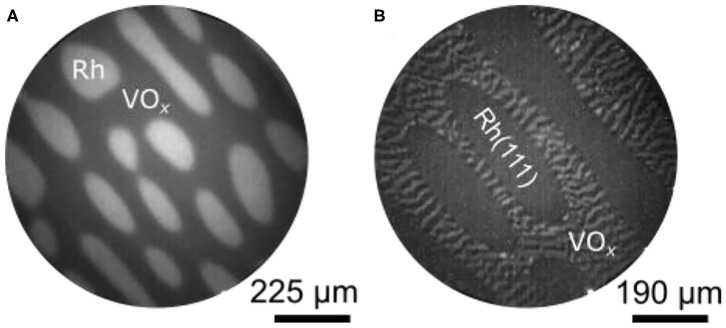
V-oxide redistribution in catalytic methanol oxidation with NO and O_2_ over VO_*x*_/Rh(111). **(A)** PEEM image showing a hole pattern during the CH_3_OH + NO reaction. (b) Change in the VO_x_ distribution as NO is exchanged against O_2_. Experimental conditions: **(A)** *T* = 1030 K, 1 × 10^−4^ mbar NO and 1.5 × 10^−4^ mbar CH_3_OH; **(B)** *T* = 1030 K, 1 × 10^−4^ mbar NO and 3 × 10^−4^ mbar CH_3_OH, θ_V_ = 0.3 MLE. Reprinted from von Boehn et al. (2018a), with the permission of AIP Publishing.

Figure 11A shows a PEEM image of the VO_*x*_/Rh(111) surface after the sample has been heated up to 1030 K in a gas mixture of CH_3_OH and NO. This pattern evolved from a pattern of thin parallel stripes existing around 900 K. As in methanol oxidation with O_2_, the VO_*x*_ covered parts of the surface appear as dark area in PEEM, the bare Rh(111) surface as bright area. In Figure 11A, holes in the VO_*x*_ layer are surrounded by VO_*x*_ covered surface. With NO a pattern forms that is inverse to the island patterns seen in the oxidation of methanol with O_2_. Such a hole pattern is also observed in ammonia oxidation with NO.

The VO_*x*_ hole pattern is rather static, compared to the circular VO_*x*_ islands, which move toward each other and coalesce in methanol oxidation with oxygen. Nevertheless, a coarsening of the VO_*x*_ depleted holes, similar to Ostwald ripening, is observed at 1030 K. This causes an elongation of the holes. Compared to the reaction with O_2_, the interface between the VO_*x*_ covered area and the bare Rh(111) surface is broad and fuzzy. As shown with LEEM, the elongated holes are aligned to atomic steps on the Rh(111) surface (von Boehn, 2020). Atomic steps are always present on a surface resulting from a slight (unavoidable) deviation of the surface orientation from the ideal cutting angle during preparation. Another striking difference with the VO_*x*_ redistribution patterns observed with O_2_ is the absence of a substructure of reduced and oxidized phases in the VO_*x*_ layer during oxidation with NO.

The different effect that NO and O_2_ have on the distribution pattern can be demonstrated nicely by exchanging the oxidizing agent NO against O_2_ after a hole pattern has already been formed. The result displayed Figure 11B shows the same surface as Figure 11A, but with a gas atmosphere switched to methanol and O_2_. Upon heating at 800 K a VO_*x*_ stripe pattern develops exclusively in the VO_*x*_ covered parts of the surface. As the stripes further coarsen, the familiar core—ring structure of a reduced core and an oxidized ring evolves around 900 K, and the stripe pattern is transformed into an island pattern at 1,000 K.

Compared to methanol oxidation with O_2_, the oxidation of methanol with NO shows a low catalytic activity on VO_*x*_/Rh(111). Since the dissociation rate of NO on the Rh(111) surface is rather high, one could have expected the opposite. The low catalytic activity might be the main reason why no detectable oxygen gradients exist in the VO_*x*_ layer and in the holes. The absence of a substructure in the VO_*x*_-layer is clearly a consequence of the absence of oxygen gradients. O-concentration profiles obtained with *in situ* μXPS during the NO + NH_3_ reaction showed that the interface VO_*x*_ covered surface/bare metal surface is rather broad and exhibits no steep gradient, quite in contrast to the oxidation with O_2_ where gradients at the interface are steep (von Boehn, 2020). This latter observation of a broad interface can be taken as evidence for a reduced line tension at the interface compared to the oxidation reaction with O_2_.

The low dynamics in the oxidation reactions with NO suggest that it is thermodynamics that is responsible for why island pattern are formed in reactions with O_2_ and hole patterns in reactions with NO. More specifically, it should be a question of the interfacial energies and of the line tension. The conclusion that the line tension VO_*x*_/metal surface is reduced in the case of NO points in this direction, but quantitative data are required in order to reach definite answers.

### Summary

Ultrathin vanadium oxide layers on Rh(111) exhibit a wealth of redistribution dynamics, ranging from the development of stripe, island and hole patterns, over oscillatory behavior to turbulent redistribution dynamics under the conditions of methanol oxidation in the 10^−4^ and 10^−2^ mbar range. Reaction-induced oxygen gradients are essential for a peculiar ripening mechanism resulting in the movement and coalescence of neighboring VO_*x*_ islands. The island movement was explained with a polymerization/depolymerisation mechanism. These oxygen gradients can also cause a phase separation inside the vanadium oxide islands, resulting in a substructure consisting of a reduced core and an outer oxidized ring. The dependence of the stability of the reduced core on the island size can lead to periodic phase transitions and simultaneous size oscillations of the vanadium oxide islands. As the total pressure is increased into the NAP regime, the core—ring structure of the VO_*x*_ islands can no longer be stabilized and turbulent redistribution is observed. Exchanging the oxidizing agent O_2_ by NO results in the formation of a hole pattern instead of a VO_*x*_ island pattern. The different redistribution patterns seen with NO and O_2_ are probably the consequence of the low catalytic activity of VO_*x*_/Rh(111) in the reactions with NO, which prevents the formation of oxygen gradients.

## Reaction Dynamics on VO_*x*_/Rh(110)

### Structure and Reactivity of VO_*x*_/Rh(110)

Characteristic for the Rh(110) surface is its enormous structural variability as evidenced by a large number of adsorbate-induced surface reconstructions studied in detail with adsorbed atomic oxygen and nitrogen (Kiskinova, 1996). Compared to the system VO_*x*_/Rh(111) that has been extremely well explored (Schoiswohl et al., 2004b, 2005, 2006), the main difference is that only a handful of studies have been performed for VO_*x*_/Rh(110). It was shown that metallic vanadium on Rh(110) has a considerable tendency to diffuse into subsurface sites forming a subsurface V/Rh alloy (Píš et al., 2013). With LEED it was demonstrated that both, V/Rh(110) and VO_*x*_/Rh(110), exhibit a number of ordered overlayer structures upon sequential deposition, but for none of these overlayers a structure model exists (von Boehn et al., 2017). Since no structure models exist, also the V coverage calibration has to be considered as tentative.

**Figure 12:**
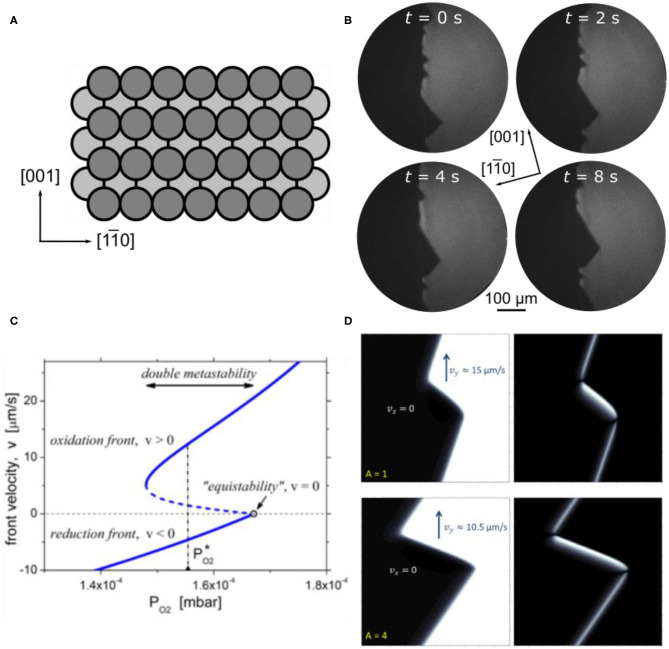
Traveling interface pulses on VO_*x*_/Rh(110) during methanol oxidation. **(A)** Sketch of the Rh(110) surface. **(B)** PEEM images showing traveling interface pulses on a Rh(110) surface promoted with some V-oxide. Experimental conditions: θ_V_ = 0.1 MLE, *T* = 820 K, *p*(CH_3_OH) = 3 × 10^**-**4^ mbar, *p*(O_2_) = 0.8 **×** 10^**-**4^ mbar. Reproduced from Ref. (von Boehn and Imbihl, 2017) with permission from the PCCP Owner Societies. **(C)** Dependence of the TIP front velocity on the oxygen pressure (1D model). The solid (dashed) lines represent the stable (unstable) solutions. In the double metastable region, oxidation and reduction fronts coexist; the dash-dotted vertical line shows the oxygen pressure at which TIPs of stationary size exist in 2D simulations. **(D)** Solitary TIP. The system size is 100 × 100 μm^2^. The color-coded spatial distributions of the oxygen coverage θ_O_ (left) and the defect concentration (right) given by difference between the oxygen coverage and the degree of reconstruction |θ_O_-θ_rec_| (right) are presented. **(C,D)** Reprinted figures with permission from Makeev and Imbihl (2019). Copyright (2019) by the American Physical Society.

In catalytic methanol oxidation VO_*x*_ on Rh(110) is far less reactive than on Rh(111), displaying only negligible activity with respect to formaldehyde production (von Boehn and Imbihl, 2018). Apparently, the system VO_*x*_/Rh(110) is more complex than VO_*x*_/Rh(111) and VO_*x*_ on the Rh(110) surface behaves quite differently than on Rh(111). Aside from a much more complex chemistry, the main new aspect that is introduced with respect to pattern formation is the anisotropy of the Rh(110) surface. A structural model of the unreconstructed Rh(110) surface in Figure 12A demonstrates this anisotropy.

### Overview

In the following, we investigate the behavior of a submonolayer coverage of V-oxide, i. e. of a V coverage in the range 0.1 ≤ θ_V_ ≤ 0.4 ML, during catalytic methanol oxidation with O_2_ in the 10^−4^ mbar range. The start is always a surface that after deposition of VO_*x*_ appears homogeneous in PEEM. It should be stressed that if the surface is homogeneous on a macroscopic scale (> 1 μm), on a microscopic scale it might be heterogeneous.

The sample is then heated up in a CH_3_OH + O_2_ atmosphere but, depending on whether we heat up above 900 K or stay below that temperature, we have a surface with VO_*x*_ islands of macroscopic size or a spatially homogeneous surface (the term homogeneous here always refers to a macroscopic length scale). Accordingly, we differentiate in the following between chemical wave patterns on a homogeneous surface and between chemical waves on a pre-patterned surface. Finally, we also consider the VO_*x*_ redistribution process itself.

### Chemical Waves on a Homogeneous Surface

#### Traveling Interface Pulses

The CH_3_OH + O_2_ reaction on the unpromoted Rh(110) surface displays bistability, i. e. one has reaction fronts separating an oxygen covered surface from a surface with a low O coverage. Close to the equistability point of the two stable surface phases, one observes a phenomenon which for the first time before has been observed in catalytic ammonia oxidation on Rh(110) (Lovis and Imbihl, 2011; Rafti et al., 2015). Distortions of the interface away from its equilibrium position travel pulse-like along the interface. These traveling interface pulses (TIPs), or traveling interface modulations (TIMs) as they were called originally, represent a front instability. A general mathematical model showing their existence has been formulated (Rafti et al., 2012), but no realistic mathematical model existed at the beginning of these studies. On a surface promoted with a low coverage of V-oxide (θ_V_ = 0.1 ML) these TIPs are much stronger in amplitude and they are much more stable than on a bare Rh(110) surface (von Boehn and Imbihl, 2017). As shown in the PEEM image in Figure 12B, waves with roughly triangular shape travel with a velocity of ~17 μm/s along the interface. Despite the anisotropy of the Rh(110) surface no clear-cut dependence of the excitations on the crystallographic directions has been found. One notes that the area after being passed by the TIPs exhibits an enhanced brightness, which then decays within a few tens of seconds. This brightness variation indicates a temporary modification of the surface either by structural defects or by a chemical modification. Since no indication for a chemical modification was found, the brightness changes were assigned to structural defects.

To explain the formation of defects around the interface, one has to keep in mind that the interface oxygen covered/oxygen free surface also represents the boundary between two different substrate structures, the so-called “missing row” reconstructions on the oxygen covered surface and the non-reconstructed (1 × 1) surface of the oxygen free surface (von Boehn, 2020). Since the two substrate structures differ in their density of surface atoms, any structural variation caused, for example, by fluctuations in the oxygen coverage, will be associated with a mass transport of Rh atoms, thus causing structural defects. A roughening of the surface caused by defects will, in general, lead to an increase in catalytic activity. This means that fluctuations in the adsorbate coverages around the interface will be amplified by a positive feedback.

The mechanism sketched above is the basis of a realistic three-variable model that semi-quantitatively reproduces well the experimentally observed TIPs (Makeev and Imbihl, 2019). As shown in the bifurcation diagram of Figure 12C, the TIPs occur in a very narrow parameter window near the equistability point in the region of so-called dynamic bistability (or double metastability) where oxidation and reduction fronts coexist. The simulation in Figure 12D shows an enhanced defect concentration in the leading edge of each pulse. TIPs occur on Rh(110) during catalytic ammonia and methanol oxidation, but TIPs have not been found in the H_2_ + O_2_ reaction. From the proposed mechanism, one should expect that TIPs should also occur in the bistable O_2_ + H_2_ reaction on Rh(110). This, however, is not the case. The mathematical model provides the explanation, which is that the region of dynamic bistability becomes vanishingly small due to the fast diffusion of adsorbed hydrogen, thus removing one of the essential requirements for the occurrence of TIPs (Makeev and Imbihl, 2019).

#### Varying Front Geometries and Traveling Wave Fragments

**Figure 13:**
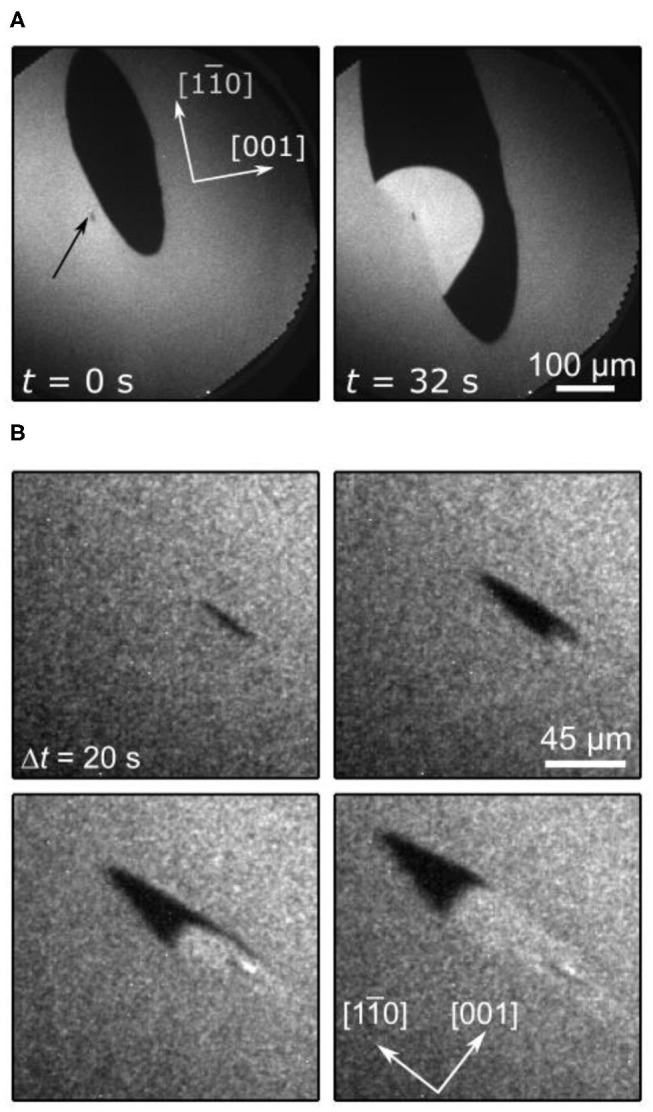
Double metastability and traveling wave fragments in methanol oxidation over VO_*x*_/Rh(110). Shown are PEEM images. **(A)** A propagating oxidation front (black) collides with a surface defect, resulting in the nucleation of a reduction front (bright). **(B)** Traveling wave fragment propagating along the [$1\bar{1}0$] direction. Field of view:173 × 173 μm^2^, Experimental conditions: *T* = 790 K **(A)** and 740 K **(B)**, *p*(O_2_) = 1 × 10^−4^ mbar, *p*(CH_3_OH) = 3 × 10^−4^ mbar, θ_V_ = 0.4 MLE. Reprinted with permission from von Boehn and Imbihl (2018). Copyright (2018) American Chemical Society.

In a coverage range 0.2 ≤ θ_V_ ≤ 0.4, we observe dynamic bistability in a temperature range that extends roughly from 700 to 850 K. As shown in Figure 13A, upon a parameter change, initially an elliptical oxygen island develops imaged as dark area in PEEM. After ≈ 20 s, a bright front nucleates inside the dark island, but this bright front, as it grows, exhibits a different anisotropy than the elliptical dark island. Apparently the anisotropy of the Rh(110) surface varies depending on the type of front. Such a behavior has before been observed in the H_2_ + NO reaction on Rh(110), and was traced back to a state-dependent anisotropy (Gottschalk et al., 1994; Mertens and Imbihl, 1994). In the case of the H_2_ + NO reaction, the different substrate reconstructions causing the state-dependent anisotropy have been identified, but for the present system no structure models exist yet.

Another phenomenon that results from a state-dependent anisotropy is also found here, which are traveling wave fragments (TWFs). Figure 13B displays PEEM images showing different stages in the development of TWFs. These TWFs typically nucleate at some defect and then propagate with constant velocity along the [$1\bar{1}0$]-direction, which is the direction along which the troughs of the Rh(110) surface (Figure 12A) are oriented. TWFs have been found before in catalytic CO-oxidation on Pt(110) (“soliton-like” behavior) (Rotermund et al., 1991), and in the H_2_ + NO reaction on Rh(110) (Mertens et al., 1995).

In Figure 13A, and less clearly in Figure 13B, we can identify three distinct gray levels—a medium gray, dark and bright—which we can assign to the resting state, the excited state and the refractory state of the surface. *In situ* LEEM is feasible in the 10^−4^ mbar range. In such a LEEM/μLEED investigation ordered overlayers could be assigned to the different phases of chemical waves on VO_*x*_/Rh(110) (von Boehn, 2020). Even with this information a mechanistic interpretation of the patterns presented above remains very difficult due to (i) a complete lack of structure models and (ii) the missing chemical information. *In situ* μXPS at synchrotrons requires a pressure < 10 ^−5^ mbar, but since the chemical waves only showed up beyond 1 × 10^−4^ mbar, the former condition excluded any *in situ* XPS study of chemical waves. Nevertheless, the similarity of the patterns observed here with the chemical wave patters seen in the H_2_ + NO reaction on Rh(110) suggests a common origin of state-dependent anisotropy, namely the switching of the surface between reconstructions with varying anisotropy.

### VO_*x*_ Redistribution Patterns

#### Island Formation

Annealing VO_*x*_ on Rh(110) in a reacting atmosphere to 1,020 K generates VO_*x*_ islands of macroscopic dimensions. Apparently, only at this temperature the mobility of VO_*x*_ on Rh(110) becomes high enough to accomplish a mass transport over macroscopic distances. At 1,020 K a VO_*x*_ island size of 100 μm is reached within minutes. Similar to Rh(111), the islands exhibit a substructure consisting of a dark outer ring and a bright core region in PEEM. Quite differently from VO_*x*_/Rh(111), however, the islands do not move under reaction conditions and they do not coalesce (von Boehn and Imbihl, 2018).

The VO_*x*_ islands respond to changes in the gas phase composition. Depending on whether they appear bright in PEEM (*p*(CH_3_OH) high) or dark (*p*(CH_3_OH) low), we speak of reduced or oxidized islands. The terms “oxidized” and “reduced” are used here on a pure phenomenological basis. In fact, the response to changes in the gas atmosphere was the main criterion to distinguish between VO_*x*_ covered areas and areas with a low VO_*x*_ coverage. Since no chemical analysis has been conducted, these assignments should best be considered as preliminary.

#### Chemical Wave Patterns

**Figure 14:**
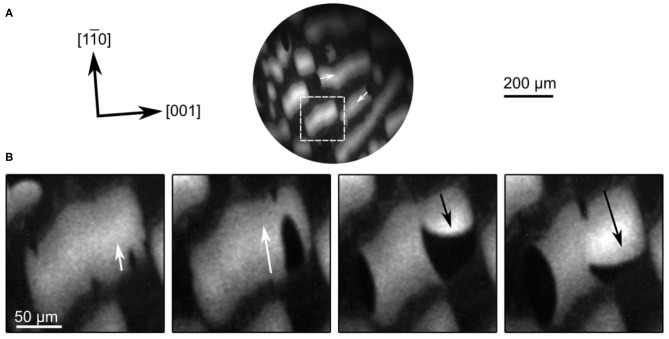
PEEM images showing traveling wave fragments propagating on VO_*x*_ islands (bright areas) during methanol oxidation on VO_*x*_/Rh(110). **(A)** Nucleation of wave fragments at the borders of the VO_*x*_ islands. The white arrows mark the propagation direction. (s) Magnified section of the images in a. Experimental conditions: T = 830 K, *p*(O_2_) = 1 × 10^−4^ mbar, *p*(CH_3_OH) = 3 × 10^−4^ mbar, Δ*t* = 10 s between frames 1–2 and 3–4, 20 s between frames 2–3 in **(B)**. Reprinted with permission from von Boehn and Imbihl (2018). Copyright (2018) American Chemical Society.

When, after preparation, such a surface with large reduced VO_*x*_ islands is heated up into the temperature window of dynamic bistability chemical wave patterns develop. As demonstrated by the PEEM images in Figure 14, TWFs nucleate and propagate across the VO_*x*_ islands. These TWFs propagate along the [$1\bar{1}0$]-direction of Rh(110), similarly to TWFs on the homogeneous surface in Figure 13B. Initially, dark elliptically shaped islands nucleate outside the VO_*x*_ islands. They invade the reduced VO_*x*_ islands acting as oxidation fronts. The oxidation fronts are soon followed by reduction fronts transforming the oxidized state into a refractory state appearing bright in PEEM. These bright reduction fronts also nucleate at the boundaries of the VO_*x*_ islands. The bright refractory state then relaxes into the resting state characterized by a medium gray level. A comparison with Figure 13B shows that the nature of the TWFs seen on the pre-patterned surface does not appear to be different from the TWFs on the homogeneous surface.

#### Incomplete Phase Separation

The process of VO_*x*_ redistribution could be followed *ex situ* with LEEM/μLEED allowing to establish correlations between the different surface phases and ordered overlayers (von Boehn et al., 2018b). The chemistry evolving in this process could not be followed *in situ* since μXPS is not feasible at 10^−4^ mbar. However, by generating an island structure at 10^−4^ mbar in a preparation chamber followed by transfer to the LEEM chamber, μXPS could at least be performed *ex situ* under UHV conditions. The data showed that different from the system VO_*x*_/Rh(111) no complete phase separation into VO_*x*_ islands and into an almost VO_*x*_ free metal surface occurred. A substantial part of VO_*x*_, roughly 15% of the signal inside the islands, is also present in the area surrounding the islands. Moreover, part of the vanadium has penetrated the Rh bulk residing in subsurface sites. These results indicate that VO_*x*_ redistribution on Rh(110) is more complex than on Rh(111).

#### Dendritic Growth

**Figure 15:**
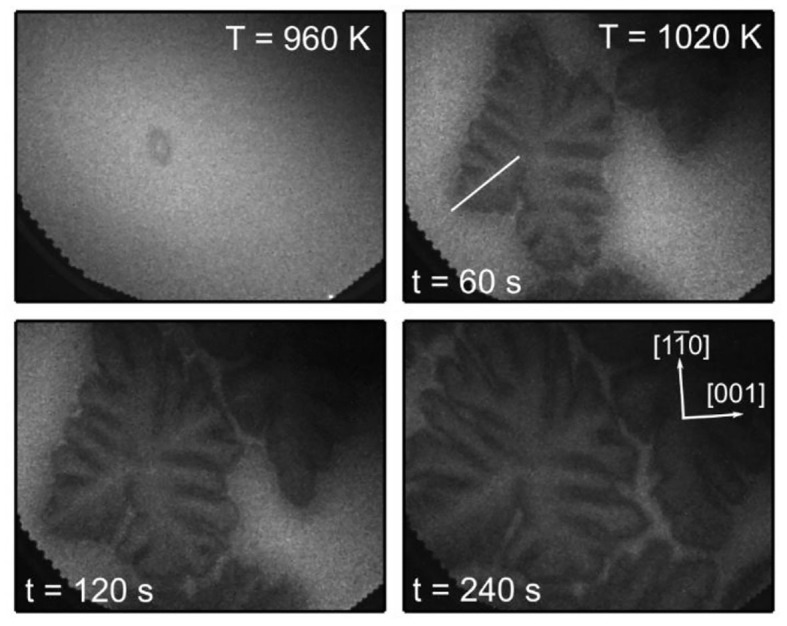
Dendritic growth of VO_*x*_ islands on Rh(110) during methanol oxidation imaged with PEEM. Experimental conditions: *p*(O_2_) = 1 × 10^−4^ mbar, *p*(CH_3_OH) ≈ 3 × 10^−4^ mbar, θ_V_ = 0.4 MLE, 500 × 400 μm^2^ field of view. Reprinted with permission from von Boehn and Imbihl (2018). Copyright (2018) American Chemical Society.

One starts with the uniform surface present in PEEM after deposition of 0.4 ML VO_*x*_. Upon heating in the CH_3_OH + O_2_ atmosphere, *p*(CH_3_OH) is varied such that the surface is kept in a reduced state, but not far from the transition to an oxidized state. In the temperature range 960–1020 K one observes nucleation and dendritic growth of the VO_*x*_ phase (von Boehn and Imbihl, 2018), as demonstrated by the PEEM images in Figure 15. Within a few minutes after the heating schedule has been stopped at 1,020 K, the whole imaged area is filled with the dendritic VO_*x*_ structure. The main axis of the dendritic structure is oriented along the [$1\bar{1}0$]-direction, which is the direction of the [$1\bar{1}0$] troughs of Rh(110). The dendritic growth of branches always stops short before they get in contact with each other such that they are still about 20 μm apart.

### Summary

The behavior of VO_*x*_ on Rh(110) in catalytic methanol oxidation is distinctly different from VO_*x*_ on Rh(111). The catalytic activity is much lower than on Rh(111) and we observe no moving and coalescing VO_*x*_ islands. Chemically and structurally the VO_*x*_/Rh(110) surface is much more complex than VO_*x*_ on Rh(111), since a population of subsurface sites by vanadium is involved. Furthermore, the complete lack of structural models for any of the ordered VO_*x*_ phases on Rh(110) complicates the interpretation of data. On the other hand, one is rewarded with a rich variety of intricate chemical wave patterns and VO_*x*_ redistribution processes.

Dynamic bistability with varying front geometries and traveling wave fragments can be attributed to a state-dependent anisotropy caused by surface reconstructions with different diffusional anisotropy. Despite the identification of numerous surface phases by μLEED/LEEM, an excitation mechanism for the chemical waves could not yet be established due to a lack of chemical information. *In situ* μXPS was not feasible under the conditions where chemical waves occur, a problem well known in catalysis under the name “pressure gap.” With further developments of surface analytical *in situ* techniques, capable of operating at elevated pressure, this obstacle should be overcome in the near future.

## Conclusion

Studying catalytic reactions on VO_*x*_/Rh(111) and VO_*x*_/Rh(110), the range of non-linear phenomena in catalysis has been expanded from metal surfaces to oxidic systems. The most significant finding for VO_*x*_/Rh(111) is an island ripening mechanism that operates under non-equilibrium conditions. This mechanism differs from classical Ostwald ripening and also from Smoluchowski ripening. The VO_*x*_ island coalescence could be explained with a polymerization/depolymerization mechanism. The system VO_*x*_/Rh(110) is more complex than VO_*x*_/Rh(111), but one is rewarded with a wealth of chemical wave patterns in addition to VO_*x*_ redistribution structures. Overall, one finds that supported V-oxide layers at submonolayer coverages are highly dynamic, giving rise to new phenomena that have not been observed on metal surfaces. The observations made here are closely related to the well-known “pressure and materials gap” in heterogeneous catalysis: structure and composition of the V-oxide layer are strongly pressure dependent. Moreover, comparing VO_*x*_ on Rh(111) and Rh(110), one finds that the catalytic properties and the pattern forming properties of the V-oxide layer vary drastically depending on the orientation of the metallic substrate. The results obtained here also demonstrate the relevance of non-linear effects in catalysis.

## Author Contributions

BB and RI wrote and edited the manuscript. All authors contributed to the article and approved the submitted version.

## Conflict of Interest

The authors declare that the research was conducted in the absence of any commercial or financial relationships that could be construed as a potential conflict of interest.

## Supplementary Material

Data Sheet 1 Short description of the videos in the supplementary material.

Supplementary Video 1 VO_*x*_ stripe and island pattern formation during methanol oxidation (PEEM).

Supplementary Video 2 VO_*x*_ island oscillation during methanol oxidation (LEEM).

Supplementary Video 3 Turbulent VO_*x*_ redistribution (NAP-LEEM).

Supplementary Video 4 Formation of a VO_*x*_ hole pattern in the CH_3_OH + NO reaction (PEEM).

Supplementary Video 5 Traveling interface modulations on VO_*x*_/Rh(110) during methanol oxidation (PEEM).

Supplementary Video 6 Traveling wave fragments on VO_*x*_ islands during methanol oxidation (PEEM).

Supplementary Video 7 Dendritic island growth on VO_*x*_/Rh(110) during methanol oxidation (PEEM).
