# Supplementary material for: Dynamics of Ultrathin Vanadium Oxide Layers on Rh(111) and Rh(110) Surfaces During Catalytic Reactions
Source: Front Chem. 2020 Aug 21;8:707. doi: 10.3389/fchem.2020.00707 (PMC7472780; doi:10.3389/fchem.2020.00707)
Supplement: Data Sheet 1 — Short description of the videos in the supplementary material. [file Data_Sheet_1.DOCX]

Supplementary Material

Dynamics of Ultrathin Vanadium Oxide Layers on Rh(111) and Rh(110) Surfaces during Catalytic Reactions

Bernhard von Boehn^1^, Ronald Imbihl^1*^

^1^ Institut für Physikalische Chemie und Elektrochemie, Leibniz Universität Hannover, Callinstraße 3A, 30167 Hannover, Germany

*** Correspondence:**Ronald Imbihl
imbihl@pci.uni-hannover.de

Keywords: Vanadium Oxide, Chemical Waves, Methanol Oxidation, Heterogeneous Catalysis, Oxide Redistribution.

Abstract

Over the past 35 years rate oscillations and chemical wave patterns have been extensively studied on metal surfaces, while little is known about the dynamics of catalytic oxide surfaces under reaction conditions. Here we report on the behavior of ultrathin V oxide layers epitaxially grown on Rh(111) and Rh(110) single crystal surfaces during catalytic methanol oxidation. We use photoemission electron microscopy and low-energy electron microscopy to study the surface dynamics in the 10^-6^ to 10^-2^ mbar range. On VO*_x_*/Rh(111) we find a ripening mechanism in which VO*_x_* islands of macroscopic size move towards each other and coalesce under reaction conditions. A polymerization/depolymerization mechanism of VO*_x_* that is sensitive to gradients in the oxygen coverage explains this behavior. The existence of a substructure in VO*_x_* islands gives rise to an instability, in which a VO*_x_* island shrinks and expands in an oscillatory manner around a critical radius. At 10^-2^ mbar the VO*_x_* islands are no longer stable but they disintegrate, leading to turbulent redistribution dynamics of VO*_x_*. On the more open and thermodynamically less stable Rh(110) surface the behavior of VO*_x_* is much more complex than on Rh(111), as V can also populate subsurface sites. At low V coverage, one finds traveling interface pulses in the bistable range. A state-dependent anisotropy of the surface is presumably responsible for intriguing chemical wave patterns: wave fragments traveling along certain crystallographic directions, and coexisting different front geometries in the range of dynamic bistability. Annealing to 1000 K causes the formation of macroscopic VO*_x_* islands. Under more reducing conditions dendritic growth of a VO*_x_* overlayer is observed.

**SM1.** PEEM video (reducing reaction conditions) showing the formation of a VO*x* stripe pattern, which evolves into a pattern of circular vanadium oxide islands, which exhibit a substructure consisting of an outer dark ring and a bright core. Experimental conditions: The sample was heated with 0.2 K/s from 870 to 1020 K. *p*(CH_3_OH) = 3⨯10^-4^ mbar, *p*(O_2_) = 1⨯10^-4^ mbar. The video is accelerated by a factor of 40. The field of view is 850 μm.

**SM2.** LEEM video showing an oscillating vanadium oxide island. The video was acquired at a start voltage of 2.0 eV in 1⨯10^-4^ mbar CH_3_OH and 1⨯10^-4^ mbar O_2_ at 1030 K. The field of view is 75 μm. The video has been accelerated by a factor of 5.7.

**SM3.** NAP-LEEM video showing VO*x* islands during the transition from a core-ring structure to turbulent dynamics. The video was acquired at a start voltage of 3.1 eV between 600 and 700 K with a field of view of 100 μm. The gas phase was composed of 1⨯10^-2^ mbar oxygen and 1⨯10^-2^ mbar methanol. The video has been accelerated by a factor of 30.

**SM4.** PEEM video showing the formation of a VO*_x_* hole pattern during the CH_3_OH + NO reaction. Experimental conditions: The sample is heated with 0.5 K/s to 1030 K, *p*(NO) = 1⨯10^–4^ mbar, p(CH_3_OH) = 1.5⨯10^–4^ mbar, the field of view is 850 µm. The video has been accelerated by a factor of 50.

**SM5.** Traveling interface modulations on VO*_x_*/Rh(110) during methanol oxidation. The video is accelerated 10 times. The video was recorded at 820 K in 3⨯10^-4^ mbar methanol and 0.8⨯10^-4^ mbar oxygen. The field of view is 650 µm. The video has been accelerated by a factor of 10.

**SM6.** Traveling wave fragments on macroscopic V-oxide islands during catalytic methanol oxidation. After the VO*_x_* island formation the sample was cooled down in a reaction atmosphere of 1⨯10^-4^ mbar oxygen and 3⨯10^-4^ mbar methanol with a constant cooling rate of 0.5 K/s. When 680 K was reached the initial dark V-oxide islands started to brighten upon reactive removal of oxygen: bright VO*_x_* islands on dark Rh. Subsequently, the sample was heated up again to 830 K and hold at this temperature. The video shows dark elliptical oxidation fronts that start to nucleate on the surrounding Rh surface propagate inside the islands. The field of view is 650 μm, the video is accelerated by a factor of 10.

**SM7.** Dendritic growth of V-oxide islands under reducing methanol oxidation conditions close to the equistabilitiy point of the oxidized and reduced state. The sample was heated up from 300 K to 1020 K with a constant heating rate of 0.5 K/s to 1020 K in 1⨯10^-4^ mbar oxygen and ≈3⨯10^-4^ mbar methanol. During heating the surface was prevented from switching into its oxidized state by carefully increasing the methanol partial pressure every time oxidation fronts started to nucleate on the surface. When 960 K were reached two VO*_x_* islands started to nucleate. The field of view is 650 μm, the video is accelerated by a factor of 10. Please note that the video is the concatenation of two datasets acquired subsequently. There is a delay of 2 - 3 minutes between the two data sets recognizable by an interruption after 20 s video time.
